# Source Separation Using Sensor’s Frequency Response: Theory and Practice on Carbon Nanotubes Sensors

**DOI:** 10.3390/s19153389

**Published:** 2019-08-02

**Authors:** Aurore Quelennec, Éric Duchesne, Hélène Frémont, Dominique Drouin

**Affiliations:** 1 Laboratoire d’Intégration du Matériaux au Système, CNRS-UMR 5218 Université de Bordeaux, 33400 Talence, France; 2 Institut Interdisciplinaire d’Innovation Technologique, Laboratoire Nanotechnologies Nanosystèmes, CNRS-UMI 3468 Université de Sherbrooke, Sherbrooke, QC J1K0A5, Canada; 3 IBM Canada Ltd., Bromont, QC J2L1A3, Canada

**Keywords:** MWCNT, source separation, dual sensor, temperature sensor, moisture sensor, gas sensor, sensing mechanism

## Abstract

Nowadays, there is an increased demand in integrated sensors for electronic devices. Multi-functional sensors provide the same amount of data using fewer sensors. Carbon nanotubes are non-selectively sensitive to temperature, gas and strain. Thus, carbon nanotubes are perfect candidates to design multi-functional sensors. In our study, we are interested in a dual humidity-temperature sensor. Here, we present a novel method to differentiate at least two sources using the sensor’s frequency responses based on multiwall carbon nanotubes sensors. The experimental results demonstrate that there are temperature- or moisture-invariant frequencies of the impedance magnitude, and their values depend on the sensor’s geometry. The proposed measurement model shows that source-invariant frequencies of the phase can be also determined. In addition, the source separation method is generalized to other materials or sources enabling multi-functional sensors for environment monitoring.

## 1. Introduction

Carbon nanotube networks are non-selectively sensitive to gas [1,2,3], moisture [4,5,6,7,8,9,10], temperature [11,12], strain [13,14], and light intensity [15]. Thus, a carbon nanotube-based sensor is inherently multifunctional. The electrical behaviour of carbon nanotube networks fits with a parallel resistor–capacitor (RC) circuit [16]. In this case, two sources of perturbations A and B can be differentiated using the frequency response of the sensor if the resistance (R) is only sensitive to A, and the capacitance (C) to B and/or A. However, previous studies [1,2,3,4,5,6,7,8,9,10,11,12] have shown that both R and C of carbon nanotube networks are sensitive to gas and temperature changes, so to extract one source response among a number of others, additional sensors or post-processing are required. Here, we will study the separation of two sources, namely temperature and moisture, using only one sensor based on multiwall carbon nanotube (MWCNT) networks in a polyimide matrix. We will present a novel method to separate two sources when the resistance and the capacitance of the sensitive material are both impacted by the quantities intended to be measured. We will show that there is a frequency where the impedance magnitude of the sensor is temperature-invariant, and thus only responds to moisture. Similarly, there is another frequency where the impedance magnitude is moisture-invariant. Finally, thanks to the sensing mechanisms and other published works, we will generalize the source separation method to other sensing materials and sources.

## 2. Methods

**Figure 1:**
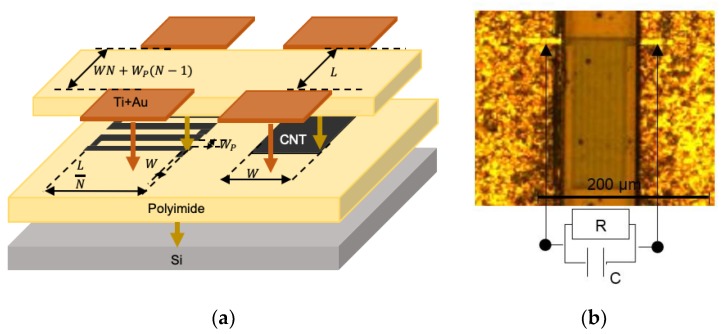
Carbon nanotube sensors. (**a**) Magnified view of the temperature-moisture sensitive layer. (**b**) Optical microscopic picture and equivalent electrical circuit of the sensor.

As shown in Figure 1a, the sensor consists of a MWCNT-based sensitive layer, either as a pad or with a serpentine geometry, connected with titanium coplanar electrodes and covered by a polyimide (PI) protective layer non-hermetic to water [17].

### 2.1. Fabrication of the Sensor

The first step was to spin-coat and cure, at 200 °C for 30 min and at 375 °C for 60 min, a 3 µm-thick polyimide surface (HD4104, HD MicroSystemsTM) on top of a bare silicon wafer. 1 µm length multiwalled carbon nanotubes functionalized with carboxyl groups (MWCNTs) were then mixed in an NMP (1-Methyl-2-pyrrolidinone, SIGMA-ALDRICH^®^, Oakville, ON, Canada) solvent following a ratio of 0.1 mg/mL. The MWCNTs were dispersed in the solution by sonication for 20 min. The resulting solution was spray-coated on top of the polyimide surface, which was placed on a hot plate at 220 °C in order to evaporate the NMP solvent. The MWCNT layer was then patterned using UV-photolithography and oxygen plasma etching techniques. The two geometries allow investigation of the length/width ratio effects, as defined in Figure 1a. The length/width ratio varies from 0.01 to 50. The serpentine geometry is used to optimize space, where N represents the number of serpentine meanders and $W_{P}$ the polyimide width between lines. Moreover, the thicknesses can vary from 400 nm to 800 nm. A top polyimide layer was added to protect the MWCNTs. The coplanar electrodes, which consist of a first 750 nm thick titanium bottom layer and a 150 nm thick gold top layer to avoid the oxidation of titanium, were deposited by e-beam evaporation and patterned by a lift-off step.

### 2.2. Measurement Procedure

With an environmental test chamber (Excal 1411-HE, Climats), the ambient environment surrounding the sensor can be controlled. The chamber’s temperature can be varied from −40 °C to 140 °C by steps of 20 °C every 20 min. Similarly, the relative humidity (RH) can be varied from 30% RH to 90% RH by steps of 10% RH every 20 min. The relative humidity can be controlled for a temperature ranging from 30 °C to 90 °C. To measure the sensor response to temperature, the chamber’s relative humidity was fixed at 30% RH. In the same way, to study the sensor response to moisture, the chamber’s temperature was fixed at 30 °C. Finally, to study the crosstalk effect of temperature and moisture on the sensor response, for each level of temperature from 30 °C to 75 °C by steps of 15 °C, the relative humidity was varied from 30% RH to 75% RH, by steps of 15% RH.

The sensor’s impedance was measured using an impedance analyser (E4990A, Keysight Technologies, Santa Clara, CA, USA). The instrument was programmed to acquire the impedance values using a two-minute sampling time. At each time step, the impedance is measured over 21 frequencies, logarithmically distributed from 20 Hz to 1 MHz.

### 2.3. Electrical Model of Carbon Nanotube Networks

The function of a carbon nanotube network is to conduct electricity between MWCNTs. Indeed, depending on the distance between MWCNTs, the carbon nanotubes can be close enough to allow the electrical conduction by tunnel effect. In case of a MWCNT or chains of MWCNTs not participating in the electrical conduction, the electrons trapped at their frontiers will polarize them. Thus, the resulting sensor is equivalent to a RC-parallel circuit, as shown in Figure 1b. The equivalent impedance ($\underline{Z}$) of the carbon nanotube network is expressed in Equation (1), as a function of the frequency (f). At low frequencies the impedance will be governed by the resistance (R) of the MWCNT mesh as at intermediate frequencies the polarization within MWCNTs will induce a capacitive (C) effect dominating the impedance. The two frequency intervals are delimited by the cutoff frequency $f_{C}=\;1/\left(2\pi \mathrm{fRC}\right)$.

(1) $$\underline{Z}\left(f\right)=\;\frac{R}{1+2\pi \mathrm{jRCf}}$$

### 2.4. Equations Governing the Sensor

Four electrical quantities of the MWCNT network are studied in the sensing mechanism: $R,\;C,\;\left|\underline{Z}\right|\;$and θ, where $\left|\underline{Z}\right|$ and θ are the impedance magnitude and the impedance phase, respectively. These quantities are considered as the sensor’s output quantity. The letter Y is used as generic name for the sensor’s output quantity. Y is characterized by its relative sensitivity $s_{Y,X}\left(f\right)$ to the source X, as expressed in Equation (2). In this study, X will be either the temperature (T) or the relative humidity (RH), and $Y_{\mathrm{ref}},{\;T}_{\mathrm{ref}},{\;\mathrm{RH}}_{\mathrm{ref}}$ are respectively the values of the output quantity, the temperature and the relative humidity at the reference operating conditions, which are 30% RH and 30 °C.

(2) $$Y=Y_{\mathrm{ref}}\left(1+s_{Y,T}\left(f\right)\left(T-T_{\mathrm{ref}}\right)+s_{Y,\mathrm{RH}}\left(f\right)\left(\mathrm{RH}-{\mathrm{RH}}_{\mathrm{ref}}\right)\right)$$

In order to compare the responses of the geometrically different sensors, the relative variation, denoted as RV, of Y is calculated as the ratio between the variation of Y and $Y_{\mathrm{ref}}$, as defined in Equation (3).

(3) $$\mathrm{RV}=\frac{\Delta Y}{Y_{\mathrm{ref}}}=\frac{Y-Y_{\mathrm{ref}}}{Y_{\mathrm{ref}}}=s_{Y,X}\left(f\right)\left(X-X_{\mathrm{ref}}\right)$$

From these equations, the sensor’s impedance magnitude is insensitive to temperature if $s_{\left|Z\right|,T}\left(f\right)=0$ or to moisture if $s_{\left|Z\right|,\mathrm{RH}}\left(f\right)=0$.

## 3. Results

The temperature and moisture measurements were performed on a same sensor, which has a serpentine geometry (N = 8, $W_{P}=2\;µm$), as defined in Figure 1a.

### 3.1. Thermoelectric Effect and Temperature-Invariant Frequency

**Figure 2:**
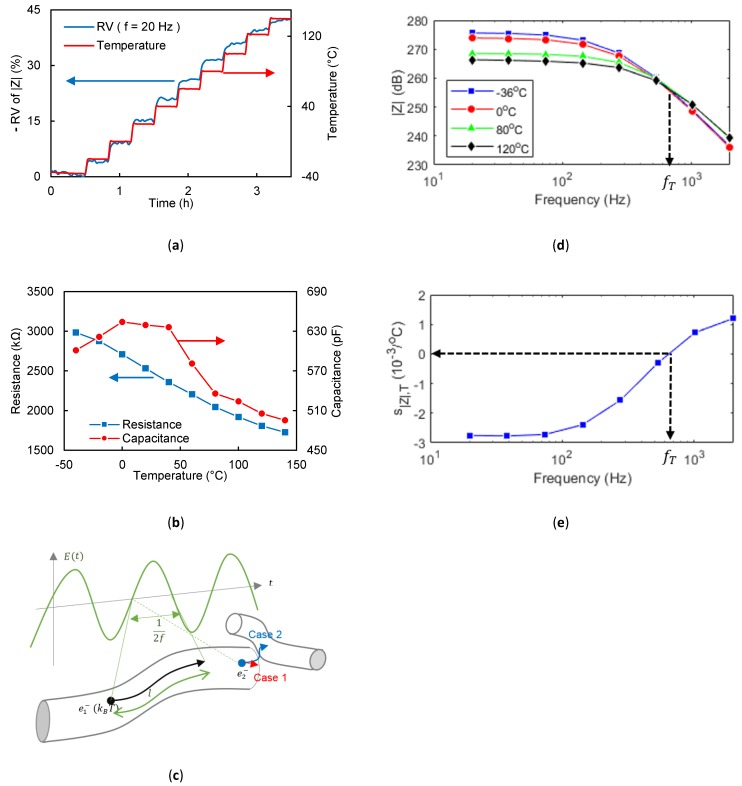
Thermoelectric effect. (**a**) Real-time low-frequency impedance magnitude-time curve during temperature cycles. (**b**) Electrical properties of multiwall carbon nanotube (MWCNT) network of different temperatures. (**c**) Multiscale schematic diagram of the sensing mechanism of the sensor with temperature changes. (**d**) Impedance magnitude of sensor of different temperatures and frequencies. (**e**) Impedance magnitude sensitivity to temperature of different frequencies.

As shown in Figure 2a, the applied temperature is incremented to quantify the relationship between the impedance and temperature. At low frequencies, like 20 Hz, $\left|\underline{Z}\right|$ declines when the temperature increases. As the behaviour is resistive on this frequency interval, R decreases when T increases, as shown in Figure 2b. Indeed, both the tunneling and the intrinsic resistance [18] of the MWCNT networks are temperature-dependent. For T≤0 °C, the capacitance increases with the temperature, but for T≥0 °C, the capacitance decreases with T. As depicted in Figure 2c, under a temperature and an alternative electrical field ($\vec{E}\left(f\right)$), an electron travels a distance l, and due to the fact that the electron speed increases with T, more electrons reach the MWCNT boundary. After reaching the interface, there are two possible outcomes: The electron goes to the nearest MWCNT, or there is an interfacial polarization of the MWCNT or the MWCNT chain [19]. Similarly, the tunneling resistance is temperature-dependent, so the transmission probability between two MWCNTs increases too. Hence, the polarization and the tunneling resistance are coupled. In fact, from the results, as long as the temperature is below 0 °C, the temperature increase helps the polarization, but over 0 °C the temperature is high enough for an electron to overpass the tunnel barrier [20,21].

(4) $$\;\exists {\;f}_{i}\left(X_{j},X_{k}\right)\;\mathrm{if}\;\left(1-{\left(\frac{R\left(X_{k}\right)}{R\left(X_{j}\right)}\right)}^{2}\right)\left(1-{\left(\frac{C\left(X_{k}\right)}{C\left(X_{j}\right)}\right)}^{2}\right)\;>0.$$

(5) $$f_{i}\left(X_{j},X_{k}\right)=\;\frac{1}{2\pi R\left(X_{k}\right)C\left(X_{j}\right)}\sqrt{\frac{1-{\left(\frac{R\left(X_{k}\right)}{R\left(X_{j}\right)}\right)}^{2}}{1-{\left(\frac{C\left(X_{k}\right)}{C\left(X_{j}\right)}\right)}^{2}}}$$

(6) $${\mathrm{For}\;T}_{k}>{\;T}_{j},{\;f}_{i}\left(T_{j},{\;T}_{k}\right)\;\sim \;\frac{1}{2\pi R\left(T_{k}\right)C\left(T_{j}\right)}$$

(7) $${\mathrm{For}\;\mathrm{RH}}_{k}>{\;\mathrm{RH}}_{j},{\;f}_{i}\left({\mathrm{RH}}_{j},{\mathrm{RH}}_{k}\right)\;\sim \;\frac{1}{2\pi R\left({\mathrm{RH}}_{j}\right)C\left({\mathrm{RH}}_{k}\right)}$$

To sum up, at low frequencies $\left|\underline{Z}\right|$ equivalent to R decreases with the temperature increase, whereas at intermediate frequencies $\left|\underline{Z}\right|$ proportional to 1/(2πCf) increases, so the temperature coefficient of $\left|\underline{Z}\right|$ is negative for low frequencies and positive for intermediate frequencies. This means that there is a frequency where $\left|\underline{Z}\right|$ is temperature-invariant. Thus, as shown in Figure 2d, there is an intersection frequency $f_{i}\left(T_{j},{\;T}_{k}\right)$ where the two impedance magnitudes of different temperatures $T_{j}$ and $T_{k}$ intersect. The intersection frequency value can be calculated by the formula defined in Equations (4) and (5). An approximation of this frequency is defined in Equation (6), using the asymptotic frequency behaviour of |Z|. The temperature-invariant frequency ($f_{T}$) is the mean of the intersection frequencies. As shown in Figure 2e, $f_{T}$ is the frequency where the relative sensitivity to temperature $s_{\left|Z\right|,T}$ is null (Equation (2)).

### 3.2. Hygroelectric Effect and Moisture-Invariant Frequency

**Figure 3:**
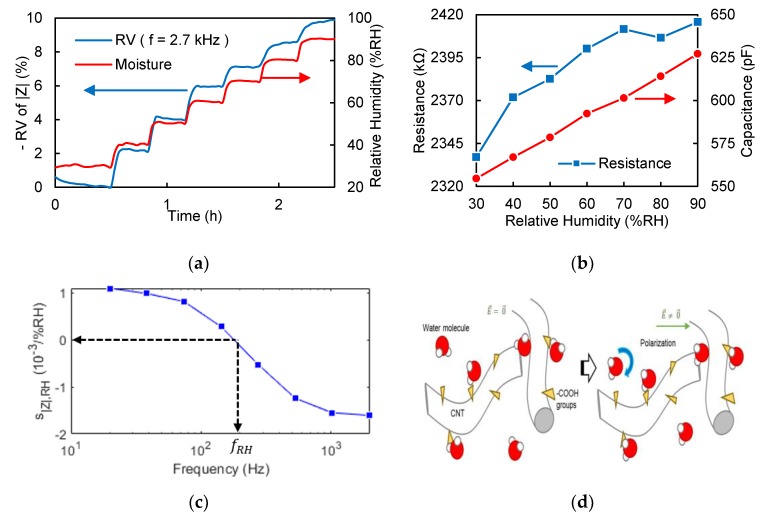
Hygroelectric effect. (**a**) Real-time intermediate-frequency impedance magnitude-time curve during moisture cycle. (**b**) Electrical properties of CNT sensor of different relative humidity values. (**c**) Relative impedance magnitude sensitivity to moisture of different frequencies. (**d**) Schematic diagram of the sensing mechanism of the sensor with moisture changes.

As shown in Figure 3a, the relative humidity is gradually increased in order to show that the MWCNT sensor also responds to moisture change. As shown in Figure 3b, R and C are increased along with number of water molecules present in the MWCNT mesh. The water molecules reduce the transmission probability between MWCNTs because they obstruct the gaps between MWCNTs, as depicted in Figure 3d.

Furthermore, water molecules are able to bind with carboxyl groups present at the MWCNT surface, inhibiting the ion conduction through carboxyl groups and explaining an increase in resistance. As water molecules are polarized, they are oriented under the alternating electrical field ($\vec{E}\left(f\right)$). The sensing mechanism is shown in Figure 3d. Hence, the increase in the number of water molecules present in the sensing material amplifies the dielectric permittivity and the capacitance (Equation (1)).

To summarize, when the level of humidity increases, the low-frequency $\left|\underline{Z}\right|$ equivalent to R increases, and the intermediate-frequency $\left|\underline{Z}\right|$ equivalent to 1/(2πfC) decreases. Therefore, the moisture sensitivity of $\left|\underline{Z}\right|$ is positive at low frequencies and negative at intermediate frequencies. In these conditions there is a moisture-invariant response frequency, written $f_{\mathrm{RH}}$, as shown in Figure 3c. Per analogy to temperature, there is an intersection frequency between two $\left|\underline{Z}\right|$ of different relative humidity levels. Its value can be extrapolated from Equations (4) and (5). An approximation of the equation is shown in Equation (7).

## 4. Discussion

### 4.1. Principle of the Temperature-Moisture Separation

In this study, we have highlighted that R and C decrease with the temperature, while R and C increase with the level of humidity. Hence, there are source-invariant frequencies if the source sensitivity of R and C have the same sign, and from Equations (6) and (7) it can be deduced that, for the same sensor, the temperature-invariant frequency is higher than the moisture-invariant frequency. In practice (Figure 2e and Figure 3d), we found that the temperature-invariant frequency is 5 times higher than the moisture-invariant frequency for the serpentine sensor studied.

As the sensor’s R and C increase for one source and decrease for the other one, leading to $f_{T}\neq f_{\mathrm{RH}}$, we formulate the hypothesis that the temperature and the moisture responses can be separate using the temperature and moisture-invariant frequencies. For a better observation of the phenomenon, another MWCNT sensor such as $f_{T}=10f_{\mathrm{RH}}$ was used. The sensor geometry is a pad and its parameters are shown on the right-hand side of Figure 1a. The impact of the temperature on the relative humidity response was studied, and vice versa. In this case, the reference temperature is 30 °C and the reference moisture is 30% RH (Equation (2)).

**Figure 4:**
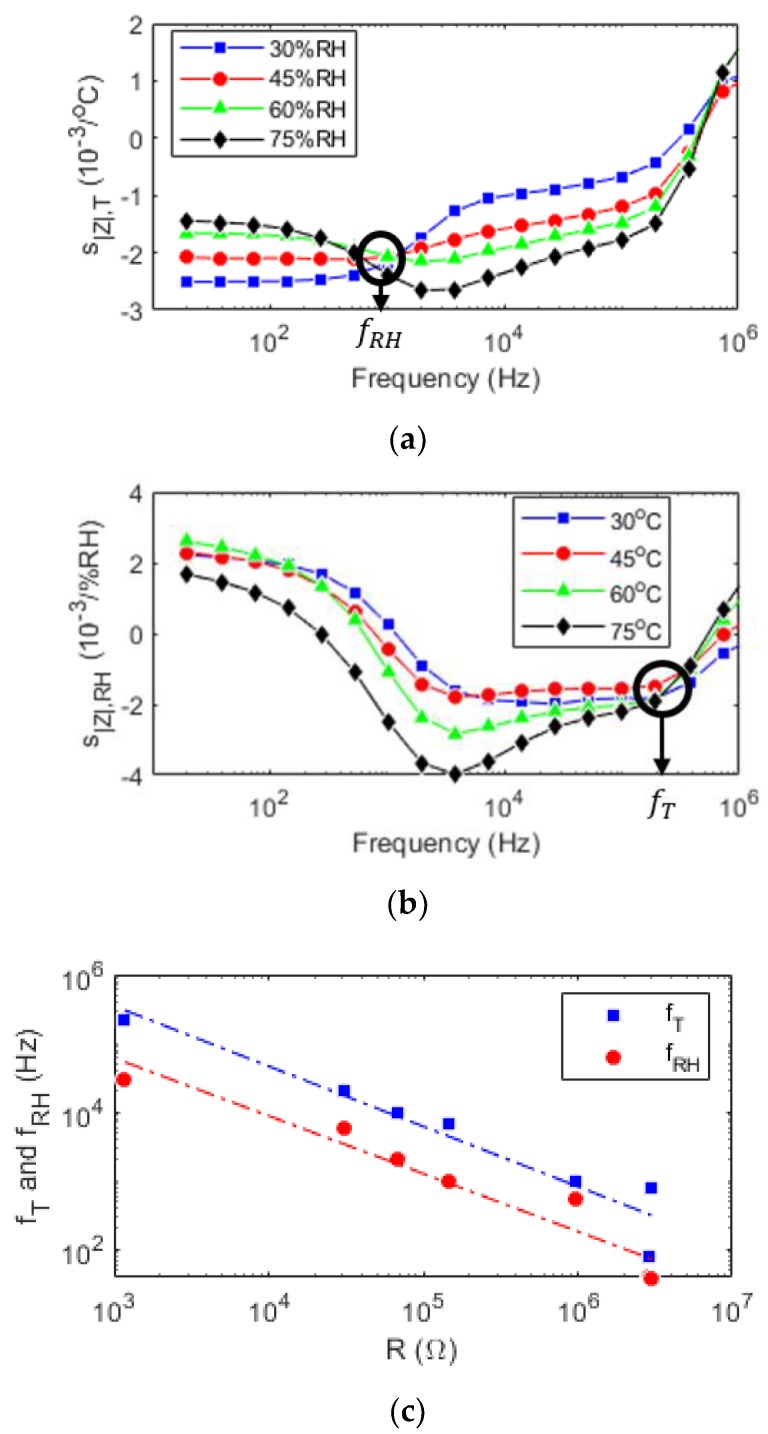
Source separation. (**a**) Relative impedance magnitude sensitivity to temperature of different moistures and frequencies. (**b**) Relative impedance magnitude sensitivity to moisture of different temperatures and frequencies. (**c**) Non-response temperature or moisture frequencies of different fabricated sensors represented via their resistance.

The frequency behaviour of the relative impedance magnitude is plotted in Figure 4a for different relative humidity values. The moisture-invariant frequency $\left(f_{\mathrm{RH}}\right)$ is determined as the intersection of the curves. At $f_{\mathrm{RH}}$, the relative sensitivity to temperature is non-zero, so when $\left|\underline{Z}\right|$ is invariant to moisture, the temperature response is isolated.

Similarly, the frequency behaviour of the relative impedance magnitude sensitivity to moisture is represented in Figure 4b for different temperatures. The curves intersect at the temperature-invariant frequency ($f_{T})$ in Figure 4b. Thus, at this frequency, $\left|\underline{Z}\right|$ only responds to moisture, and its sensitivity is non-zero, so the moisture response can be decoupled from the temperature response at the temperature-invariant frequency$\;(f_{T})$.

To conclude, temperature and moisture changes are discerned using respective source-invariant frequencies of the $\left|\underline{Z}\right|$ variation of the sensor.

In addition, because the reference resistance value is proportional to the geometric parameters $R_{\mathrm{ref}}=\rho L/\mathrm{We}$ and because the source-invariant frequency value is proportional to R (Equations (6) and (7)), two sensors with different geometries can have identical temperature and moisture-invariant frequencies, as shown in Figure 4c. Therefore, using two different sensors’ geometries and the same frequency, the temperature and moisture responses can also be decoupled.

Other results [1,2,3,4,5,6,7,8,9,11,12,22,23] confirm the sign of the temperature and moisture coefficient of resistance and capacitance of carbon nanotube networks. The behaviour is similar for carbon nanotubes, whether functionalized or not [23], for microscale or macroscale [23] sensors and for multiwall or single-wall [5] carbon nanotubes.

### 4.2. Extension to Other Sources or Technologies of Sensors

If the source sensitivity of R and C have the same sign, it can be extrapolated from Equation (4) that there is a source-invariant frequency, so there is source-invariant frequency for other sensing materials or sources. Using published works, we will extend the proposed sensing mechanism and differentiation method to other sources or sensing materials.

In the presented sensing mechanism, other polarized particles that can bind to the carbon nanotube surface should be detected and have respective particle-invariant frequencies. As example, ammonia molecules are polarized and are able to bind to functional carbon nanotubes through a hydrogen bond. Thus, equivalent resistance [24] and capacitance [3] increase with elevated ammonia concentration in the ambient environment. Therefore, there is an ammonia-invariant frequency, which is visible in Figure 5 from [16].

Ferrite nanocrystals have source-invariant frequencies as well. For instance, ZnFe_2_O_4_ [25] resistance and capacitance are reduced by the increment of temperature. Thus, ZnFe_2_O_4_ has a temperature-invariant frequency. Moreover, in this research paper, Zn_0.5_Ni_0.5_Fe_2_O_4_ shows a 200 °C Curie temperature, so its capacitance increases below this temperature and decreases otherwise while its resistance continuously declines with the increase of temperature. Thus, above 200 °C the response ensures a temperature-invariant frequency.

The use of ceramics as sensing elements to detect moisture or temperature is often used in micro-devices. Their resistance and capacitance increase with temperature [26]. Therefore, there is a temperature-invariant frequency for ceramic-based sensors. The moisture response can then be extrapolated at this frequency. Resistance and capacitance variation signs to moisture change are opposite, which implies that there is no moisture-invariant frequency. The relative sensitivity of the resistance ($s_{R,\mathrm{RH}}\left(f\right)$) to moisture is equivalent in value to the capacitance ($s_{C,\mathrm{RH}}\left(f\right)$) one. Thus, in this case, the phase is insensitive to moisture, as shown in Equation (8).

(8) $$\tan\left(\theta \right)-\tan\left(\theta_{\mathrm{ref}}\right)\sim -\;2{\pi \mathrm{fR}}_{\mathrm{ref}}C_{\mathrm{ref}}\;\left(s_{R,\mathrm{RH}}\left(f\right)+s_{C,\mathrm{RH}}\left(f\right)\right)\Delta \mathrm{RH}$$

The temperature is measured using the sensor’s phase. Hence, if the source sensitivity of resistance and capacitance have different signs, there are source-invariant frequencies of the phase.

### 4.3. Selection of the Output of Dual Sensors

Depending on the sign of $s_{R,X}$ and $s_{C,X}$ of two Sources A and B, we sum up in a truth table (Table 1) which electrical parameter of the sensor may be studied. Indeed, the last two columns report if the resistance, capacitance, impedance magnitude or phase at a source-invariant frequency measure directly the source A or B. $f_{A}$ and $f_{B}$ are respectively the A and B-invariant frequencies, and “PP” denotes that the output requires post-processing. In the first four columns, the source A/B sensitivity of R/C can be either positive (+), negative (−) or null (0). Not all situations are covered here, first because sources A and B are switchable. Then, for all other cases, the proposed method is not useful, and both R and C need post-processing to separate the sources A and B.

## 5. Conclusions

We have demonstrated that there are source-invariant frequencies of the impedance magnitude or phase, allowing the source separation at these different frequencies. We have applied this method to decouple temperature and moisture responses on carbon nanotube-based sensors. In fact, the impedance magnitude of carbon nanotube-based sensors exhibits a temperature-invariant frequency and a second moisture-invariant one. These frequencies can be determined semi-empirically, knowing the sensitivity of the resistance and capacitance to the sources. In addition to common methods to decouple two sources that are limited to sensors, which have a resistive response to a source and a capacitive response to the other one, we provide methods when sources act on both resistance and capacitance.

## 6. Patents

Carbon Nanotube-Based Multi-Sensor

Publication number: 20180231484; Publication date: 16 August 2018

Abstract: Carbon nanotube-based multi-sensors for packaging applications and methods to form the carbon nanotube-based multi-sensors are capable of simultaneously measuring at least two measurands including temperature, strain, and humidity via changes in its electrical properties.

Inventors: Eric Duchesne, Dominique Drouin, Hélène Frémont, Simon Landry, Aurore F.M.E. Quelennec, Umar Shafique, Patrick R.J. Wilson

## Figures and Tables

**Table 1:** Truth table for the selection of the output electrical parameter.

$s_{R,A}$	$s_{C,A}$	$s_{R,B}$	$s_{C,B}$	Source A	Source B
+/−	0	0	+/−	R	C
−	−	+	0	C	PP on R
		0	+	R	$\left|\underline{Z}\left(f_{A}\right)\right|$
		+	+	$\left|\underline{Z}\left(f_{B}\right)\right|$	$\left|\underline{Z}\left(f_{A}\right)\right|$
+	+	−	0	C	PP on R
		0	−	R	$\left|\underline{Z}\left(f_{A}\right)\right|$
0	+/−	+	−	$\theta \left(f_{B}\right)$	R
		−	+	$\theta \left(f_{B}\right)$	R
